# Mediators of Inflammation and Immune Responses in the Human Gastrointestinal Tract

**DOI:** 10.1155/2013/865638

**Published:** 2013-09-15

**Authors:** E. Arranz, A. S. Peña, D. Bernardo

**Affiliations:** ^1^Mucosal Immunology Laboratory, IBGM, Universidad de Valladolid—CSIC, Valladolid, Spain; ^2^Laboratory of Immunogenetics, Department of Medical Microbiology and Infection Control, VU University Medical Centre Amsterdam, Amsterdam, The Netherlands; ^3^Antigen Presentation Research Group, Imperial College London & St. Mark's Hospital, Harrow HA1 3UJ, UK

The gastrointestinal tract is continuously exposed to foreign antigens—mainly derived from the commensal microbiota and food antigens—but occasionally to those derived from invading bacteria, viruses, and tumoral antigens. Therefore, the immune system of the gut has a unique capacity to balance the mechanisms of tolerance in health and those creating a proper defensive immune response in disease. Changes in such delicate balance are usually linked to the development of gastrointestinal pathology. Despite its central role in human health and disease, most of the current knowledge of mucosal immunology of the gastrointestinal tract is mainly obtained from experimental murine models. Although the mechanisms of intestinal immunity in mouse and human have similar output, the specific pathways through which they are elicited are different [1, 2]. It is essential to fill in this gap in our current knowledge of the human immune system of the gastrointestinal tract in order to understand the pathogenesis and be able to design rational therapies to manage acute and chronic inflammatory gastrointestinal disease. In this special issue, we aimed to gain depth into the current understanding of immune processes in the human gastrointestinal tract in health and disease by selecting work in progress of active investigators in the field.

L. Pastorelli et al. review the role of a new cytokine IL-33, member of the IL-1 family, in promoting host defense against parasite and involved in the pathogenesis of ulcerative colitis. The authors discuss some contradictory reports on IL-33 function in the gastrointestinal mucosa, where it has been reported either to enhance inflammatory responses or to promote epithelial integrity [3, 4]. 

Significant advances have been achieved in the understanding of the pathogenesis of inflammatory bowel disease (IBD), and new therapy targets are cytokines as well as their receptors and signaling pathways [5]. In this issue, F. Scaldaferri et al. review the several immune factors taking part in inflammatory bowel disease and how they are modulated in the course of therapy aiming to identify potential targets to control. These authors have confidence that new emerging techniques, like microarray analysis or miRNA analysis, which are able to assess immune signatures in response to therapy, could help to identify good candidates for mucosal prognostic biomarkers, together with new therapeutic targets for future research.

Vascular endothelial growth factor (VEGF) is a predominant angiogenic factor, and recent studies using tissue microarray blocks of VEGF-A, VEGF-C, VEGFR-2, and VEGFR-3 expression have shown an association to progression, invasion, and metastasis leading to poorer survival rates and prognosis [6]. G. Karamanolis et al. show an increased expression of both VEGF and CD31 in postradiation rectal biopsy specimens, suggesting that the blockage of VEGF may represent a therapeutic option in patients with these severe conditions that is refractory to available therapies.

Recent works suggest that in coeliac disease both the innate and the acquired immune response are involved in the inflammation initiated and maintained by gluten and key to the development of autoimmunity in this common disease [7]. A small fraction of patients become refractory to the gluten-free diet, the only current available treatment [8, 9]. Recent studies suggest that it is more frequently observed in Europe than in the United States [10]. It is characterized by persistent or recurrent symptoms of malabsorption and intestinal villous atrophy. In this issue, S. Gross et al. have studied refractory CD type II (RCDII), a particular subtype with extreme bad prognosis and in fact it is considered a low-grade intraepithelial lymphoma [11]. Gross et al. have found that IL-13 may play a key role as a proinflammatory cytokine since it is correlated with IL-17A production and to other TH1 and TH2 cytokines, but not to the regulatory cytokine IL-10, thus confirming their hypothesis that the immune response is differentially regulated by cytokines in active coeliac disease versus RCDII. This finding opens new mechanisms to study further in order to understand the pathogenesis of this condition.

Obesity, the modern epidemics, is according to a new vision associated with chronic low-grade inflammation, and the intestinal microflora may be responsible for inducing these changes [12]. Since fecal microbiota transplantation re-establishes a balanced intestinal flora with resultant cure of recurrent clostridium difficile infection [13], other conditions may benefit from this approach. In this issue, R. Mehta et al. have studied gene expression profile in gastric tissue of morbidly obese patients with different histological forms of nonalcoholic fatty liver disease (NAFLD) and have identified an altered profile for several inflammatory molecules. This finding may be responsible for the pathogenesis of obesity-related NAFLD. Previously, it had been demonstrated that alterations in intestinal microbiota are associated with obesity and six weeks after infusion of microbiota from lean donors increased insulin sensitivity of recipients along with levels of butyrate-producing intestinal microbiota. Thus suggesting that intestinal microbiota might be developed as therapeutic agents to increase insulin sensitivity in humans, however, increased knowledge of the intestinal microbiota in health maintenance as well as controlled trials of fecal microbiota transplantation is needed before it can be accepted to be used clinically [14, 15].

The activation of several cytosolic pathogen recognition receptors allows the assembly of the inflammasome, a multimeric complex platform that leads to the activation of the innate immune system [16]. In their paper presented in this issue, T. Nunes and H. S. de Souza review our current knowledge on the inflammasome. The known molecular structure, its importance in maintaining intestinal homeostasis, and its critical mechanisms of the inflammasome are described in the context of chronic inflammatory disorders in the human gut such as inflammatory bowel diseases (IBD) and intestinal cancer. 

M. Witkowska and P. Smolewski, in this issue, review the role of *Helicobacter pylori* infection in chronic inflammation and the subsequent genomic transformation and development. Knowledge on the etiology, pathogenesis, treatment, and follow-up of gastric mucosa-associated lymphoid tissue (MALT) lymphoma is providing more insight into mechanisms of inflammation of this infection that is fortunately continuously decreasing in the western world. *Helicobacter pylori* eradication is a first-line treatment of gastric MALT lymphoma; however, a significant percentage of patients do not respond to treatment. Recently, it has been found that a high number of Treg cells or a high ratio of Treg cells to the total number of CD4+ T cells in gastric MALT lymphoma could predict responsiveness to eradication therapy [17].

Manipulation of gut microbiota composition by using probiotics is being explored as a promising avenue of prophylactic and therapeutic intervention against gut inflammation. Current evidence provides support for the consideration of probiotics therapy for intestinal diseases, keeping in mind that efficacy of probiotics is strain and disease specific. The variety of studies carried out with distinct strains of probiotics bacteria has suggested heterogeneous and strain-specific effects. Because of the limitations of most studies conducted with probiotics, with regard to the power of the study, deficit of human studies, randomization, use of different strains, and lack of standardized methodology, it remains difficult to draw firm conclusions from the current trials [18]. In this issue, R. Sengupta et al. discuss the role of cell surface-associated molecules in the probiotics and their host receptor. These mechanisms will help to have a better understanding of the probiotics-host crosstalk and contribute to improve therapies to treat or prevent gastrointestinal inflammation in IBD.

Nonsteroidal anti-inflammatory drugs (NSAIDs) are the most highly prescribed drugs in the world for the treatment of pain, inflammation, and fever. However, these drugs produce serious gastrointestinal complications during long-term administration, particularly in the elderly [19]. M. Sinsa et al., in this issue, describe the action of NSAIDs as a cause of morbidity/mortality related to gastric and duodenal ulcer disease and discuss different approaches to prevent or minimize such adverse effects. However, they caution that these treatments are effective to some extent, but most of them are also associated with other risks, and there is a need to develop novel therapeutic agents to make the use of NSAIDs safer.

Particular probiotics of the *Lactobacillus* species appear to stimulate health promoting effects in the gastrointestinal tract, such as pathogen inhibition by competing with invading bacteria, immunomodulation, and enhancement of the epithelial integrity [20] not only via direct contact but also through bacteria derived metabolites [21]. Recent works suggest that dendritic cells (DC) control the nature and location of immune response that seems to play a central role in ulcerative colitis [22]. Dendritic cells may orchestrate the abnormal response against the commensal microbiota that is present in these patients [23]. E. R. Mann et al. describe an abnormal phenotype and function of circulating DC in patients suffering from ulcerative colitis which are partially restored by the probiotic strain *Lactobacillus casei Shirota.*

It has been a pleasure to select the work presented in these areas by experts in the respective fields. We hope that their findings will help to enrich the knowledge of the mediators of inflammation of the human gastrointestinal tract and will form the basis for new approaches to the treatment of common infections and those conditions that although rare have such a bad prognosis.

*E. Arranz* *E. Arranz* *A. S. Peña* *A. S. Peña* *D. Bernardo* *D. Bernardo*
